# Trastuzumab‐based near‐infrared photoimmunotherapy in xenograft mouse of breast cancer

**DOI:** 10.1002/cam4.5302

**Published:** 2022-10-18

**Authors:** Susumu Yamashita, Miho Kojima, Nobuhiko Onda, Toshinori Yoshida, Makoto Shibutani

**Affiliations:** ^1^ Laboratory of Veterinary Pathology, Division of Animal Life Science Institute of Agriculture, Tokyo University of Agriculture and Technology Tokyo Japan; ^2^ Innovation and Core Technology Management, Olympus Corporation Tokyo Japan; ^3^ Cooperative Division of Veterinary Sciences, Graduate School of Agriculture Tokyo University of Agriculture and Technology Tokyo Japan; ^4^ Institute of Global Innovation Research Tokyo University of Agriculture and Technology Tokyo Japan

**Keywords:** breast cancer, human epidermal growth factor receptor 2 (HER2), near‐infrared photoimmunotherapy, trastuzumab, IRDye700DX

## Abstract

Near‐infrared photoimmunotherapy (NIR‐PIT) is a novel form of cancer treatment using conjugates of antibody against overexpressed antigens in cancers and photoabsorber IRDye700DX. HER2 is overexpressed in various cancers, for which molecular targeted therapy such as trastuzumab has been developed. The present study investigated the efficacy potential of HER2‐targeted NIR‐PIT using trastuzumab‐IRDye700DX conjugate (Tra‐IR700) in HER2‐positive breast cancer. We first examined the reactivity of Tra‐IR700 and the cytotoxicity of NIR‐PIT in vitro*.* HER2‐positive BT‐474 and SK‐BR‐3 cells and HER2‐negative BT‐20 cells were used. Tra‐IR700 fluorescence was only observed in HER2‐positive breast cancer cell lines, and the fluorescence was localized to the cell surface. Furthermore, HER2‐positive breast cancer cell lines treated with NIR‐PIT showed swelling and blebbing shortly after irradiation, and eventually increased PI‐positive dead cells. Next, tumor accumulation of Tra‐IR700 and tumor damage by NIR‐PIT were examined in vivo. Tra‐IR700 was administered intravenously to a xenograft model in which BT‐474 cells were implanted subcutaneously in BALB/c nude mice. Tra‐IR700 fluorescence was the highest in tumor tissue 1 day after administration, and the fluorescence was localized to the cell membrane of tumor cells. At this time point, NIR‐PIT resulted in diffuse necrosis of tumor tissues 1 day after irradiation. These results suggest that NIR‐PIT with Tra‐IR700 induces a highly selective therapeutic effect in a HER2‐positive breast cancer model. NIR‐PIT using Tra‐IR700 is expected to be a novel treatment for HER2‐positive cancers, including breast cancer.

## Graphical abstract

In the present study, the applicability of Trastuzumab‐based NIR‐PIT in HER2‐positive breast cancer was examined. As a result, we found Tra‐IR700 accumulates BT‐474 xenograft tumors and the tumors were induced diffuse necrosis by NIR‐PIT.

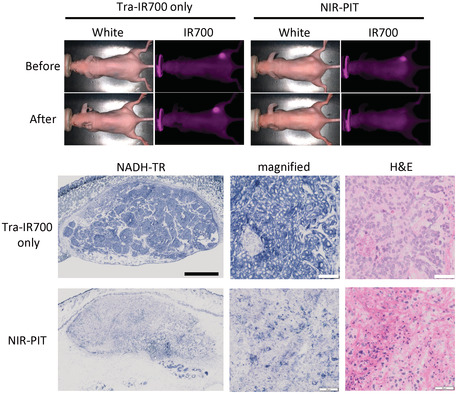

## 1 INTRODUCTION

Near‐infrared photoimmunotherapy (NIR‐PIT) is a novel form of cancer treatment that employs an antibody photoabsorber conjugate (APC) and a device for NIR light irradiation in combination.^1^ An APC consists of a monoclonal antibody (mAb) against cancer antigens and a photoabsorber, IRDye700DX (IR700), covalently conjugated to the antibody.^2^ The APC binds to cancer antigen overexpressed at the cell membrane and the irradiation induces rapid cell death with NIR light at approximately 690 nm.^3^, ^4^, ^5^ A worldwide phase 3 clinical trial of ASP‐1929, an anti‐epidermal growth factor receptor (EGFR) antibody cetuximab conjugated with IR700, is ongoing in patients with recurrent or inoperable head and neck cancer (NCT03769506). In Japan, ASP‐1929 was conditionally approved for clinical use in September 2020 as a first‐in‐class APC.^6^, ^7^ To expand the utility of NIR‐PIT, it is necessary to develop APCs targeting other tumor‐specific antigens and investigate the availability of APCs to various cancers in pre‐clinical studies.

Human epidermal growth factor receptor 2 (HER2) is a receptor tyrosine‐protein kinase erbB2, also known as CD340, proto‐oncogene Neu. It is a transmembrane glycoprotein encoded by the *ERBB2* gene. *ERBB2* has been reported to be amplified or overexpressed in clinical cancers, 15%–30% of mammary, 10%–30% of stomach including gastroesophageal junction and other cancers, such as those of the lung, ovary, bladder.^8^ As an antibody against HER2, trastuzumab is the most frequently used for HER2‐positive breast cancer. It is a recombinant humanized mAb that binds to the extracellular domain IV of HER2, and approved as antibody drug by the FDA in 1998 to treat patients with metastatic breast cancers overexpressing HER2. Thus, HER2 is a useful tumor‐associated antigen, and some antibodies have been clinically applied, making it a highly suitable target for NIR‐PIT. Previously, several studies using trastuzumab‐based NIR‐PIT have been reported in animal models of lung, ovarian, gastric, and bladder cancer.^3^, ^4^, ^5^, ^9^

The present study investigated NIR‐PIT using trastuzumab‐IR700 conjugate (Tra‐IR700) to treat a mouse model of breast cancer overexpressing HER2. Using three human breast cancer cell lines overexpressing or lacking HER2, the in vitro binding capability of Tra‐IR700 to the cell membrane and the cytotoxic effects of Tra‐IR700‐mediated NIR‐PIT were examined. Next, tumor tissue accumulation and intratumoral distribution of Tra‐IR700 were examined using tumor‐bearing mice. Finally, the in vivo effects of NIR‐PIT on the tumor tissues were examined.

## 2 MATERIALS AND METHODS

### 2.1 Reagents

The photoabsorber, silica‐phthalocyanine derivative, IRDye700DX NHS ester and IRDye700DX carboxylate were purchased from LICOR Biosciences. IRDye700DX NHS ester was used for trastuzumab conjugation, and IRDye700DX carboxylate was used for the examination of IR700 alone. Trastuzumab (Herceptin) was purchased from Chugai Pharmaceutical.

### 2.2 Synthesis of IR700‐conjugated antibodies

Trastuzumab was conjugated with IR700 according to a previous report,^1^ with some modification. Briefly, 6.8 nmol of trastuzumab was incubated with 25 nmol of IR700 in 0.1 M Na_2_HPO_4_ (pH 8.5) at room temperature for 1 h. Purified Tra‐IR700 was obtained by an ultrafiltration column (Nanosep 30 K; Pall Life Sciences) to remove unconjugated IR700. To confirm the protein concentration and the binding of three IR700 molecules on average to a single antibody, absorption at 280 and 689 nm was measured by a spectrometer (NanoDrop One; Thermo Fisher Scientific). The protein concentration and the number of IR700 molecules per antibody molecule were calculated following the manufacturer's protocols.

### 2.3 Cell culture

Three human breast cancer cell lines, BT‐20, SK‐BR‐3 and BT‐474, were used in this study. BT‐474 and SK‐BR‐3 are HER2‐positive cell lines, and BT‐20 is an HER2‐negative cell line.^10^ These cell lines were purchased from the American Type Culture Collection. BT‐20 was cultured in minimum essential medium (Thermo Fisher Scientific), SK‐BR‐3 cells were cultured with McCoy's 5A medium (Thermo Fisher Scientific) and BT‐474 cells were cultured in Dulbecco's modified Eagle's medium (Thermo Fisher Scientific). Each culture medium was supplemented with fetal bovine serum (Thermo Fisher Scientific), 10% (v/v) for BT‐20 and SK‐BR‐3, 20% (v/v) for BT‐474 respectively, in addition to 1% (v/v) penicillin/streptomycin (Thermo Fisher Scientific). Cells were incubated at 37°C in a 5% CO_2_ humidified atmosphere.

### 2.4 Fluorescence imaging systems

Live cell images were captured using a fluorescence inverted microscope (IX‐73; Olympus Corporation) or a fluorescence virtual slide scanning system (VS120‐FL; Olympus Corporation). VS120‐FL was also used for tissue section imaging. Macroscopic images of mice were captured using an in‐house NIR fluorescence imaging system consisting of excitation light by LEDs at a peak of 660 nm (SMBB660‐1100‐02; Ushio Opto Semiconductors) and a monochrome camera (GS3‐U3‐15S5M‐C, Point Grey Research) equipped with a 692‐nm long‐pass filter (FF01‐692/LP‐25‐D; Semrock). All fluorescence images were converted to pseudo‐colors by cellSens Dimension software (Olympus Corporation) or Image J software (National Institutes of Health). The analysis of fluorescence images was performed by Image J software.

### 2.5 Live cell imaging

Cells were seeded onto 8‐well chamber slides and incubated for 24 h. Culture medium was replaced with fresh medium containing Tra‐IR700 (10 μg/ml) or IR700 (1 μM), and incubated for 1 h at 37°C. Then, nuclei staining was performed using Hoechst 33342 (Thermo Fisher Scientific), followed by washing twice with PBS.

### 2.6 In vitro NIR‐PIT

Cells were seeded onto 96‐well plates and incubated for 24 h. Culture medium was replaced with fresh medium containing Tra‐IR700 (10 μg/ml) or IR700 (1 μM), and incubated for 1 h at 37°C. Then, NIR‐light irradiation was performed to the cells using a 690‐nm continuous wave laser (BWF1‐690‐300‐E; B&W TEK) at an energy density of 16 J/cm^2^, without changing the medium. The power density was measured with a radiometer (PD300‐BB‐50 mW; OPHIR Photonics).

### 2.7 In vitro cytotoxicity assay

After irradiation, morphological changes of cells were subjected to time‐course imaging by phase‐contrast microscopy. The cytotoxic effects on cultured cells in response to NIR‐PIT were also determined by Hoechst/propidium iodide (PI) double‐staining and the cytotoxicity lactate dehydrogenase (LDH) assay. For Hoechst/PI double‐staining, 1 day after NIR‐PIT, cells were incubated with fresh medium containing PI (500 nM; Thermo Fisher Scientific) and Hoechst (5 μg/ml) for 10 min at room temperature, followed by washing twice with PBS. Hoechst‐ and PI‐positive cells were counted in images of the same region. At least 100 Hoechst‐positive cells were counted per image. The cell death ratio was calculated by the PI/Hoechst‐positive cell ratio. LDH assay was performed 1 day after NIR light irradiation using a Cytotoxicity LDH Assay Kit‐WST (Dojin Chemicals). Absorbance at 490 nm was measured by a microplate reader (SH‐8000; Corona Electric), and the cytotoxicity was calculated following the manufacturer's protocols.

### 2.8 Animal model

Nude mice (BALB/c‐nu; female; age, 4 weeks) were purchased from Charles River Laboratories Japan, and acclimatized for 1 week. The mice were inoculated subcutaneously with 1 × 10^7^ BT‐474 cells suspended in a 1:1 mixture of medium and Matrigel (356237; Corning). Tumor volume was calculated using the following formula: tumor volume = length × width^2^ × 0.5. Tumors were studied after they reached volumes of 40–114 mm^3^. All animal experiments in the current study were performed in accordance with the Guidelines for Proper Conduct of Animal Experiments, (Science Council of Japan, 1 June 2006) and with the protocol approved by the Animal Care and Use Committee of The Tokyo University of Agriculture and Technology (approval no.: 30‐103, 30‐136). All efforts were made to minimize animal suffering.

### 2.9 In vivo and cellular distribution analysis of administered Tra‐IR700

Mice were administered with Tra‐IR700 at 300 μg/mouse intravenously via retro‐orbital vein. In vivo time‐course fluorescence images were obtained after the administration, and the fluorescence images of each time‐point were analyzed. Regions of interests (ROIs) were placed on tumor and left dorsum region (non‐tumorous region opposite to tumor region as background) of the fluorescence images with a white light image as the reference. The mean fluorescence intensity of each ROI was measured, and target‐to‐background ratios (TBR) were calculated by dividing fluorescence intensities of tumor by fluorescence intensities of background. Euthanasia of the mice was performed by exsanguination under deep isoflurane anesthesia within 2 days after the administration. Resected tumor tissues were snap‐frozen in liquid nitrogen. Unfixed frozen sections were subjected to observation for the cellular distribution of administered Tra‐IR700, then fixed with methanol. Fixed frozen sections were immunostained with the primary and secondary antibodies listed in Table S1, followed by counterstaining with DAPI (Thermo Fisher Scientific).

### 2.10 In vivo NIR‐PIT

BT‐474 tumors were randomized into three groups: (i) Control, no treatment (*n* = 2); (ii) Tra‐IR700 only, i.v. injection of Tra‐IR700 without NIR light irradiation (*n* = 3); and (iii) NIR‐PIT, i.v. injection of Tra‐IR700 by NIR light irradiation (*n* = 10). NIR light irradiation was performed 1 day after the injection of Tra‐IR700 under isoflurane anesthesia with a 690‐nm continuous wave laser (ML7710‐690; Modulight) at an energy density of 100 J/cm^2^. Fluorescence images of IR700 before and after NIR light irradiation were obtained. ROIs were placed on the tumor region of the fluorescence images. The mean IR700 fluorescence intensity each ROI was measured. The fluorescence intensity ratio, which is the rate of change in IR700 fluorescence by NIR‐light irradiation was calculated by dividing IR700 fluorescence intensity after NIR light irradiation by IR700 fluorescence intensity before NIR light irradiation.

### 2.11 Histological analysis

Euthanasia of the mice treated with NIR‐PIT was performed by exsanguination via the abdominal aorta under deep isoflurane anesthesia. Tumor tissues were resected 1 day after NIR light irradiation, which has been reported in a histological analysis,^11^ and divided into two pieces. One was fixed with 10% formalin for at least 24 h, and the other was snap‐frozen in liquid nitrogen. Paraffin‐embedded sections were stained with H&E. We measured the damaged area of neoplastic cells characterized by cell swelling or necrosis on H&E‐stained tumor tissue sections. Damaged areas larger than 100 μm were subjected to drawing in H&E images blinded to NIR‐PIT treatment information using Photoshop Elements 2019 (Adobe Systems). Tumor damage was calculated as a percentage of the damaged area in the total tumor area. Nicotinamide adenine dinucleotide‐tetrazolium reductase (NADH‐TR) staining was performed to identify tumor tissue damage in parallel with the assessment of H&E staining. Staining solution was prepared by adding 0.024 g β‐NADH and 0.03 g nitro blue tetrazolium to 30 ml Tris–HCl buffer (pH 7.4). Cryosections were placed in staining solution for 30 min at 37°C, and then washed and mounted.

### 2.12 Statistical analysis

Numerical data consisting of more than two sample groups were assessed for significant differences using one‐way ANOVA followed by Tukey's test. Numerical data consisting of two sample groups were analyzed for homogeneity of variance by F‐test. When the variance was homogeneous, Student's *t*‐test was applied. When the variance was heterogeneous, Aspin–Welch's *t*‐test was applied.

## 3 RESULTS

### 3.1 In vitro characterization of Tra‐IR700 in three breast cancer cell lines

**FIGURE 1:**
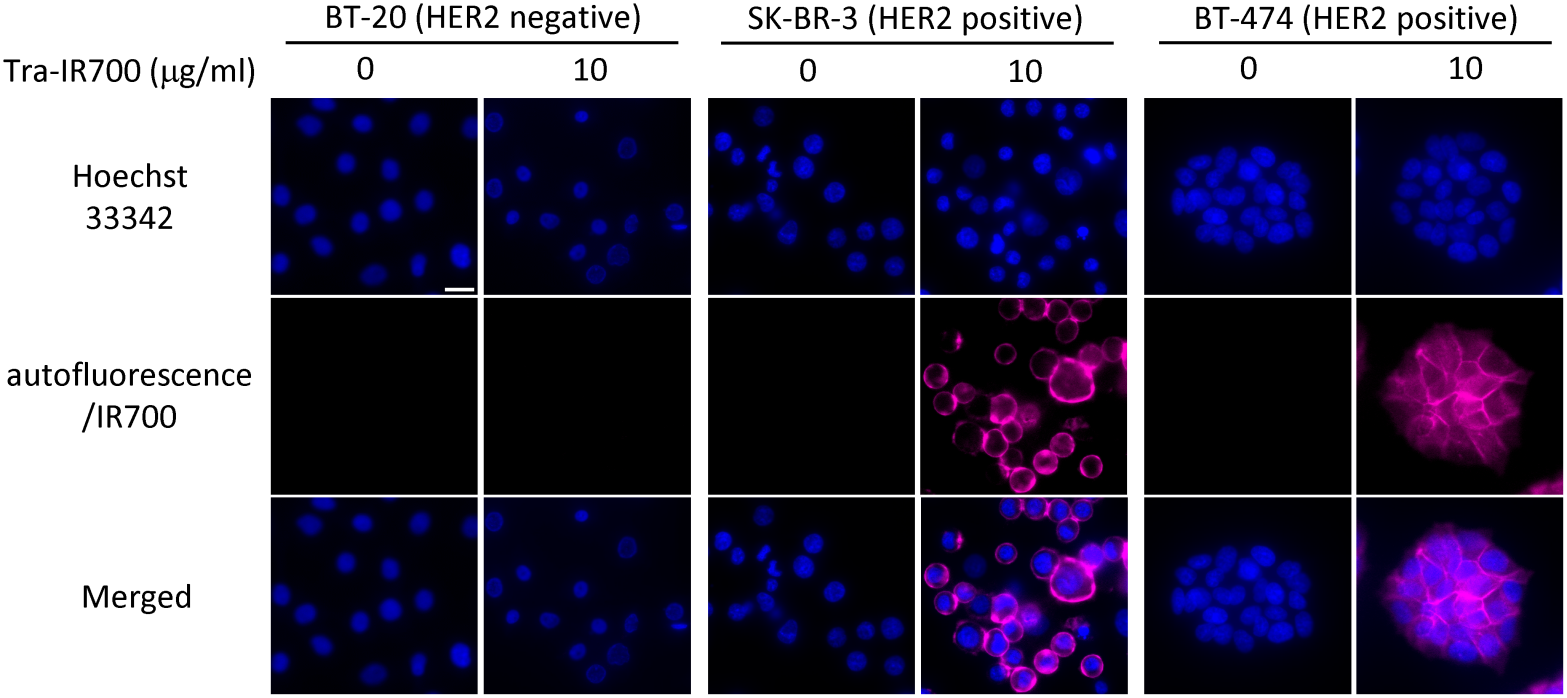
Tra‐IR700 localization in three breast cancer cell lines in vitro. Fluorescence images of three breast cancer cell lines. Tra‐IR700 fluorescence was observed in SK‐BR‐3 and BT‐474 cells. These fluorescence signals were localized on the cell surface. Fluorescence of Tra‐IR700 was not observed in BT‐20 cells. Image at 0 μg/ml indicates that of autofluorescence signal. Scale bar = 20 μm.

The binding capability and target specificity of Tra‐IR700 in three breast cancer cell lines were analyzed by fluorescence microscopy. These cell lines did not show autofluorescence signal under IR700 observation condition. After incubation with Tra‐IR700 for 1 h, SK‐BR‐3 and BT‐474 cells showed cell surface localization of Tra‐IR700 (Figure 1). SK‐BR‐3 and BT‐474 cells without Tra‐IR700 showed no fluorescence. In contrast, a fluorescence signal was not observed in BT‐20 cells incubated with Tra‐IR700 (Figure 1). In addition, a fluorescence signal was also not observed in BT‐20 and SK‐BR‐3 cells treated with IR700 (Figure S1).

### 3.2 In vitro NIR‐PIT using Tra‐IR700 in breast cancer cell lines

**FIGURE 2:**
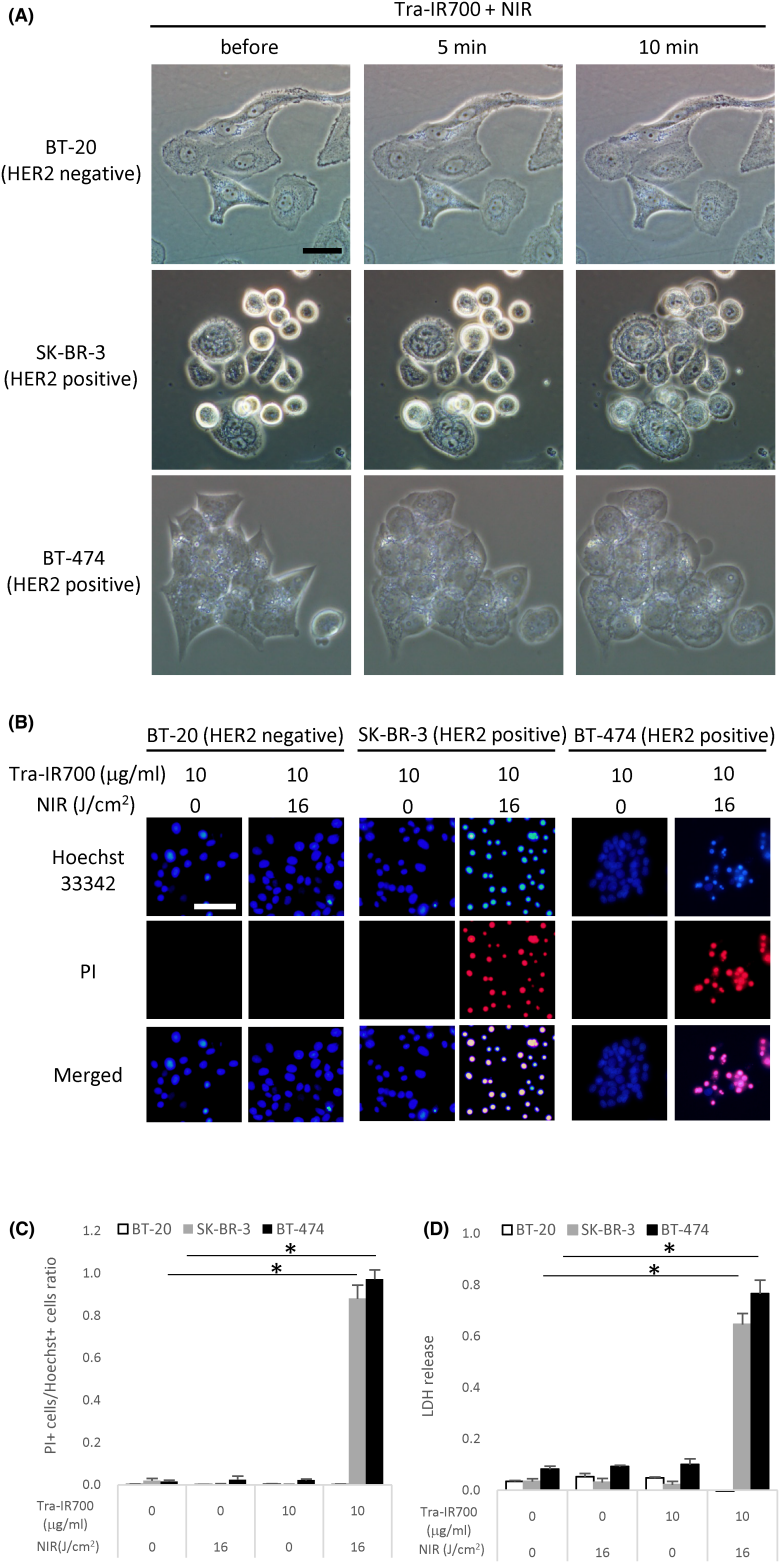
Evaluation of cell damage by NIR‐PIT with Tra‐IR700 in three breast cancer cell lines in vitro. (A) Phase contrast images before and after NIR light irradiation in three breast cancer cell lines. Tra‐IR700‐treated SK‐BR‐3 and BT‐474 cells showed morphological changes after NIR light irradiation. Scale bar = 20 μm. (B) Double‐staining images of Hoechst and PI 1 day after NIR light irradiation. NIR‐PIT‐treated SK‐BR‐3 and BT‐474 cells showed high PI signals, while BT‐20 and NIR‐PIT‐untreated SK‐BR‐3 and BT‐474 cells did not show PI signals. Scale bar = 50 μm. (C) Cell death ratio measured by calculating PI‐positive cells/Hoechst‐positive cells. Data are presented as means ± SD (*n* = 4; one‐way ANOVA followed by Tukey's test; *p < 0.001). (D) LDH assay in three breast cancer cell lines. LDH release was increased in NIR‐PIT‐treated SK‐BR‐3 and BT‐474 cells. Data are presented as means ± SD (*n* = 4; one‐way ANOVA followed by Tukey's test; *p < 0.001).

The morphological changes of cultured cells after NIR‐PIT were observed by phase‐contrast microscopy. BT‐474 and SK‐BR‐3 cells treated with Tra‐IR700 showed swelling 5 min after irradiation and blebbing on the cell membrane 10 min after irradiation, while these morphological changes were not observed in BT‐20 cells (Figure 2A). BT‐20 and SK‐BR‐3 cells treated with IR700 did not show obvious morphological changes after irradiation (Figure S2A). Hoechst/PI double‐staining showed that the number of PI‐positive dead cells was increased in BT‐474 and SK‐BR‐3 cells 1 day after NIR‐PIT, in comparison with cells without irradiation (Figure 2B). The cell death ratio in BT‐474 cells and SK‐BR‐3 was 0.97 ± 0.04 and 0.88 ± 0.06, respectively (Figure 2C). However, SK‐BR‐3 treated with IR700 did not show an increase in PI‐positivity 1 day after irradiation (Figure S2B). In addition, LDH assay revealed that cell membrane damage was induced in BT‐474 and SK‐BR‐3 cells by NIR‐PIT (Figure 2D). There was no difference in the Hoechst/PI double‐staining and LDH assay among the non‐treated, NIR light irradiation alone, and Tra‐IR700 treatment alone groups (Figure 2C,D). BT‐20 cells did not show cell death or cell membrane damage after NIR‐PIT using Tra‐IR700 (Figure 2B–D).

### 3.3 In vivo kinetics of Tra‐IR700 in BT‐474 tumor‐bearing mice

**FIGURE 3:**
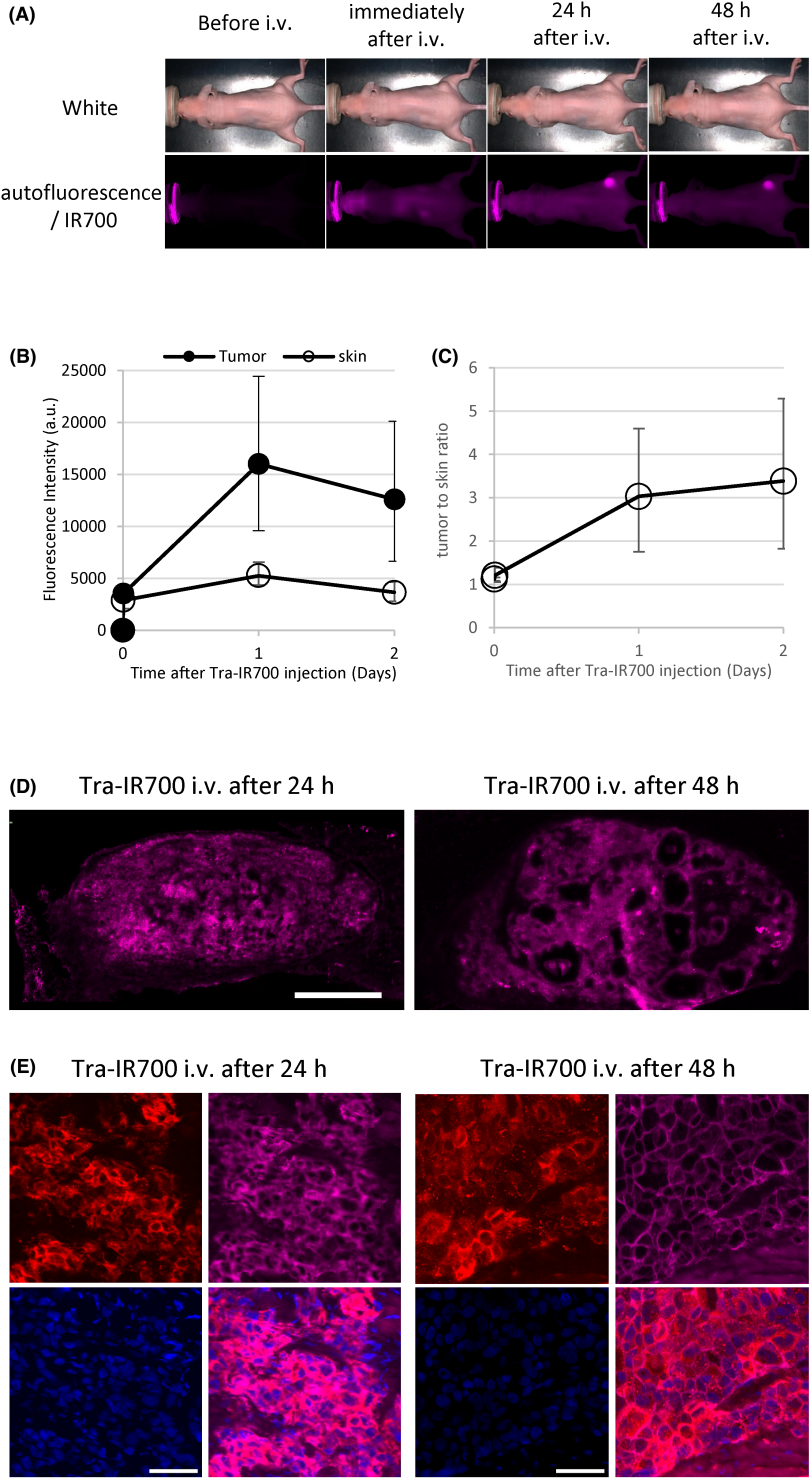
Fluorescence imaging of administered Tra‐IR700 in BT‐474 tumors. (A) Time‐course imaging of in vivo Tra‐IR700 fluorescence in BT‐474 tumor‐bearing mice (right dorsum). Before i.v. image indicates that of autofluorescence signal. (B‐C) Time course of changes in the fluorescence signal intensity of Tra‐IR700 and target‐to‐background ratio (TBR) in tumors. Data are presented as the means, and the horizontal bars indicate maximum and minimum values (*n* = 3). (D) Tissue distribution of Tra‐IR700 in BT‐474 tumors 1 or 2 days after injection. Scale bar = 500 μm. (E) Cellular distribution of Tra‐IR700 in BT‐474 tumors 1 or 2 days after injection. Upper left, HER2 (red) immunostaining in tissue sections by antibodies binding to different epitopes from those recognized by Tra‐IR700. Upper right, tissue immunostaining for trastuzumab (magenta) using secondary antibody against human IgG in the same regions as the left panel. Lower left, DAPI (blue) in the same regions as above. Lower right, composite image of HER2 (red), Tra‐IR700 fluorescence (magenta) and DAPI (blue). Scale bar = 50 μm.

BT‐474 tumor‐bearing mice showed high HER2 immunoreactivity throughout the tumor tissues (Figure S3). In vivo time‐course imaging showed that the fluorescence of Tra‐IR700 in BT‐474 tumors was increased 1 day after administration and decreased the next day (Figure 3A,B). In addition, BT‐474 tumor‐bearing mice before Tra‐IR700 administration did not show any autofluorescence signal under IR700 observation condition. The TBR of Tra‐IR700 was not clearly changed between 1 and 2 days after administration (Figure 3C). In the tumor tissues, the fluorescence signal of Tra‐IR700 was homogeneously distributed (Figure 3D). At the cellular level, the signal of intravenously injected Tra‐IR700 was mainly localized to the cell membrane of tumor cells (Figure 3E). The distribution pattern of Tra‐IR700 was not obviously changed between 1 and 2 days after administration.

### 3.4 In vivo NIR‐PIT using Tra‐IR700 in BT‐474 tumor‐bearing mice

**FIGURE 4:**
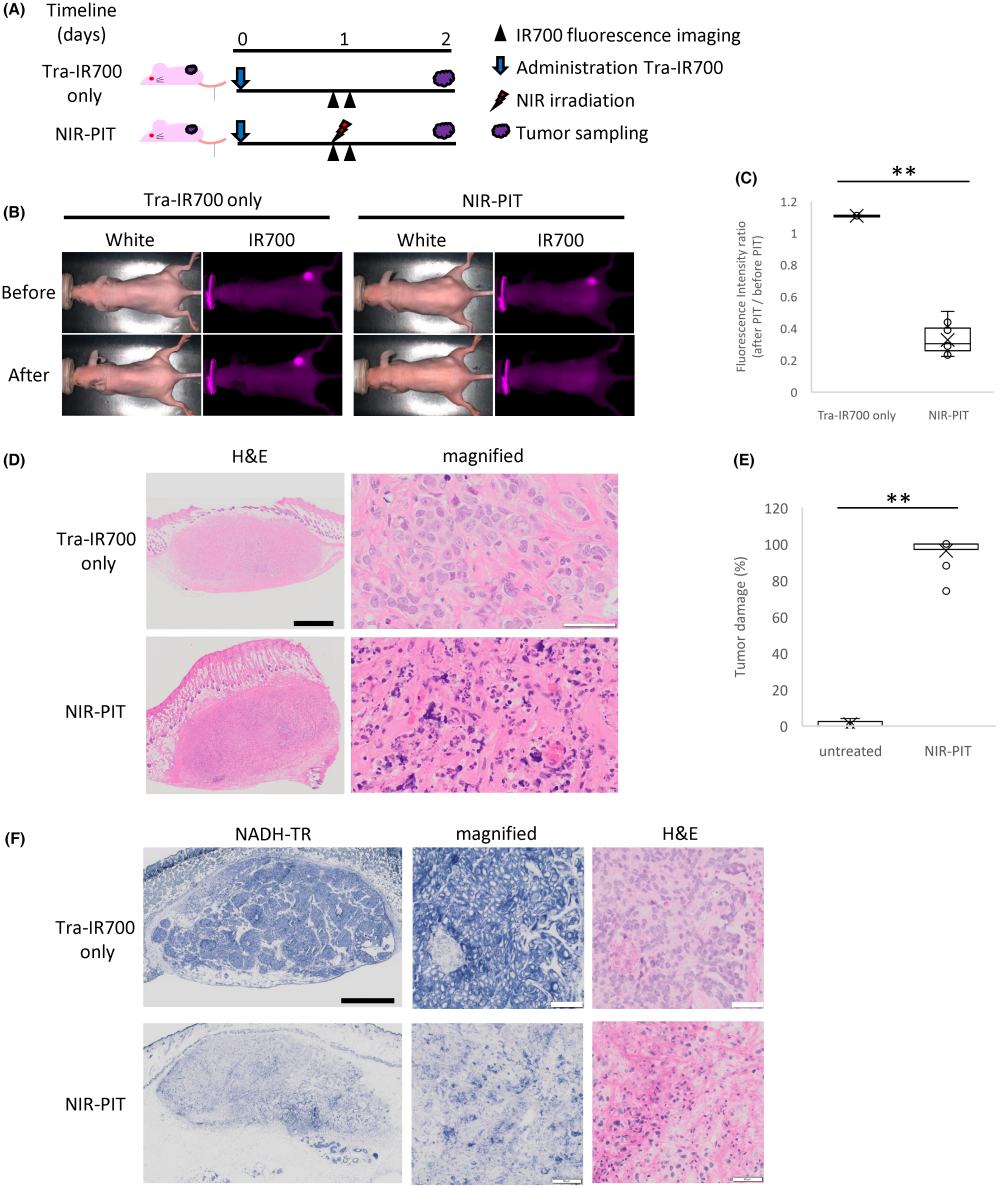
Evaluation of NIR‐PIT effects in BT‐474 tumor model. (A) The regimen of NIR‐PIT in vivo. Fluorescence images were obtained at each time point as indicated. (B) Time‐course imaging of in vivo Tra‐IR700 fluorescence in BT‐474 tumor‐bearing mice in response to NIR‐PIT. Tumors treated with NIR‐PIT showed decreased Tra‐IR700 fluorescence just after NIR light irradiation. Upper‐left panel uses same data as shown in Figure 3A (right‐middle panel). (C) Intensity ratio of Tra‐IR700 fluorescence before and after NIR‐PIT treatment. The NIR‐PIT‐treated group showed significantly decreased Tra‐IR700 fluorescence compared with the Tra‐IR700 only group (*n* = 3–10; Aspin–Welch's *t*‐test; **p < 0.05). (D) Histological images of resected BT‐474 tumor 1 day after NIR‐PIT. In the NIR‐PIT group, tumor cells showed diffuse cellular necrosis and micro‐hemorrhage. Black scale bars = 1 mm. White scale bars = 50 μm. (E) Quantification of damaged areas of tumor tissue by NIR‐PIT. The damaged areas of tumor tissue were scored by percentage (%) in histological images. In the NIR‐PIT group, the tumor tissue damage was significantly increased compared with the NIR‐PIT‐untreated group (*n* = 5–10; Aspin–Welch's *t*‐test: **p < 0.05). (F) NADH‐TR‐stained images of BT‐474 tumor sections 1 day after NIR‐PIT. In the NIR‐PIT group, NADH staining was lacking. Black scale bars = 1 mm. White scale bars = 50 μm.

One day after administration of Tra‐IR700, NIR irradiation to BT‐474 tumor tissues was performed (Figure 4A). In the NIR‐PIT group, the fluorescence signal of Tra‐IR700 was significantly reduced compared with the Tra‐IR700 only group (Figure 4B,C). Tumor tissues treated with NIR‐PIT showed necrotic changes associated with nuclear pyknosis and fragmentation, eosinophilic cytoplasm, and micro‐hemorrhage, while tumor tissues of the Control and Tra‐IR700 only groups untreated with NIR‐PIT presented a viable and highly cellular tumor (Figure 4D). These necrotic changes were observed throughout the tumor tissues of 2–3 mm in diameter. Histological analysis revealed that the damaged tissue area was significantly increased in the NIR‐PIT group (96.2 ± 8.6%) compared with the NIR‐PIT untreated group (1.0 ± 1.8%; Figure 4E). In the NADH‐TR staining, tumor tissues treated with NIR‐PIT were negatively stained, while those untreated with NIR‐PIT were almost completely positively stained. The areas of NADH‐TR negatively‐stained tissues were matched with damaged tumor regions characterized by nuclear pyknosis and eosinophilic cytoplasm in H&E staining, and the areas of NADH‐TR positively‐stained tissues were matched with intact regions in H&E staining (Figure 4F).

## 4 DISCUSSION

In the current study, we examined the reactivity of Tra‐IR700 in three breast cancer cell lines, BT‐20, SK‐BR‐3, and BT‐474. Tra‐IR700 bound to the cell membrane of HER2‐positive cell lines, SK‐BR‐3 and BT‐474, while the HER2‐negative cell line, BT‐20, did not show Tra‐IR700 binding. These results indicate that Tra‐IR700 can bind specifically to HER2 protein on the cell membrane of cancer cells, consistent with previous reports using Tra‐IR700 in other cell lines.^1^, ^12^, ^13^ A previous study suggested that a conjugate of photoabsorber with antibody that bound specifically to cell surface membrane led to necrotic cell death through a photochemical reaction.^14^ In the present study, HER2‐positive cells treated with Tra‐IR700 revealed rapid morphological changes through NIR light irradiation. These cells showed swelling and then blebbing 5 and 10 min after irradiation, respectively. In addition, double‐staining of Hoechst and PI showed that NIR‐PIT using Tra‐IR700 induced cell death in HER2‐positive breast cancer cell lines 1 day after irradiation, and the LDH assay results suggested that this cell death was induced by cell membrane damage. The formation of blebs following cell swelling has been reported as a typical feature of necrotic cell death,^15^ and the obtained results in this study suggest that NIR‐PIT induces cellular necrosis following damage to the cell membrane, leakage of intracellular components out of the cell, and formation of blebs. Cytotoxicity was not induced in the absence of Tra‐IR700 and without NIR light irradiation, suggesting that NIR‐PIT cytotoxicity requires both the presence of APCs and NIR irradiation. However, cytotoxicity by NIR‐PIT was not induced in BT‐20 HER2‐negative breast cancer cells. In addition, cells treated with IR700 alone did not show cellular uptake of IR700 regardless of HER2 expression, and the IR700‐treated cells did not show morphological changes or increases in PI‐positive dead cells after NIR irradiation. These results suggest that targeting of tumor cells using antigen–antibody binding is necessary for induction of NIR‐PIT‐mediated cell death. Photodynamic therapy (PDT) is an existing technology for cancer treatment using photosensitizers and light. In PDT, however, it has been reported that extracellular photosensitizers can induce a cytotoxic effect in normal cells.^16^ In contrast, in this study, unbound Tra‐IR700 or IR700 was not washed out before NIR irradiation but did not show cell death. These results suggest that extracellular Tra‐IR700 or IR700 did not contribute to cytotoxicity, in contrast to PDT‐mediated cytotoxicity. In summary, NIR‐PIT with Tra‐IR700 shows highly specific cytotoxicity only by Tra‐IR700 bound to the cell membrane in vitro.

In vivo imaging revealed that the fluorescence signal of Tra‐IR700 in grafted BT‐474 subcutaneous tumors was the highest 1 day after administration in consistent with previous report using Tra‐IR700.^17^ At this time point, administered Tra‐IR700 was localized to the BT‐474 tumor cell membrane, suggesting maximal binding of administered Tra‐IR700 to HER2 molecules on the surface of BT‐474 tumor cells. Therefore, the optimal time point for in vivo NIR light irradiation to induce therapeutic effects was determined to be 1 day after administration of Tra‐IR700. In the present study, 1 day after NIR‐PIT, BT‐474 tumors showed necrotic changes associated with nuclear pyknosis and fragmentation, and eosinophilic cytoplasm and micro‐hemorrhage. These results were supported by the result of NADH‐TR staining, in which dark‐blue‐stained regions reflecting cell viability were decreased by NIR‐PIT. However, the NIR‐PIT‐untreated group did not show tumor damage. Thus, the obtained results suggest that HER2‐targeted NIR‐PIT can specifically damage tumor cells in the current model of HER2‐positive cancers. In addition, histological analysis showed diffuse necrosis throughout tumor tissues of 2–3 mm in diameter. These results suggest that tumor in the subcutis of 2–3 mm thickness is capable of undergoing treatment under the conditions of NIR‐PIT in this study. A previous report demonstrated that the irradiation light employed in NIR‐PIT penetrates approximately 50% of a 3‐mm‐thick chicken breast.^18^ In this study, 100 J/cm^2^ of NIR light was irradiated, and thus, 50 J/cm^2^ was estimated to reach the deepest point of the tumor. This dose is sufficient to induce cytotoxicity based on the in vitro results showing that 16 J/cm^2^ irradiation was sufficient to kill cancer cells. In the present study, we also observed that Tra‐IR700 fluorescence was significantly decreased in tumors treated by NIR light irradiation, suggesting that photobleaching of Tra‐IR700 fluorescence was induced by NIR light irradiation. These results were consistent with a previous report demonstrating that photobleaching is caused by a photochemical reaction to release the axial‐ligand of IR700 when exposed to NIR light.^14^ Therefore, the reduction in fluorescence is common regardless of the type of APCs, and measurement of photobleaching may be useful to predict the cytocidal effect of the photochemical reaction by NIR‐PIT, as shown in previous reports.^19^, ^20^, ^21^

This study has several limitations. First, the effects of NIR‐PIT were examined only 1 day after irradiation in the present study. Because the present study was aimed at demonstrating the efficiency of HER2‐targeted NIR‐PIT in HER2‐positive breast cancer, we had to fix the time point of the histological assessment of tumors after irradiation and we could demonstrate diffuse necrosis 1 day after NIR‐PIT. Second, the grafted human cancer model used in this study was created using immunodeficient mice. However, the therapeutic effect on the tumor should take into account the activation of the immune response, including cancer immunity. Furthermore, NIR‐PIT is also known to cause indirect effects on tumor tissue through activating anti‐tumor immunity by immunogenic cell death.^22^ Therefore, evaluation of the therapeutic effects in immunodeficient models may only provide the direct effect of NIR‐PIT on cancer cells. To estimate its clinical application, the overall anti‐tumor effect of NIR‐PIT should be evaluated including the additional effect by immune activation. It may be desirable to evaluate the overall NIR‐PIT effect in a syngeneic mouse model harboring cancer cells overexpressing human HER2, as established previously in a human EGFR overexpression model.^23^

In the current study, we revealed the effectiveness of NIR‐PIT using Tra‐IR700 in a HER2‐positive breast cancer model. HER2 acts as an important biomarker and thereby as a target of cancer therapy for approximately 30% of breast cancer patients. The current standard therapy for HER2‐positive breast cancers includes chemotherapy in combination with trastuzumab.^24^ However, the efficiency of this HER2‐targeted therapy is diminished because of the primary and acquired resistance of HER2‐positive tumors to trastuzumab treatment.^25^, ^26^ Previously, it has been reported that HER2‐targeted NIR‐PIT functions well in cultured cells of HER2‐positive breast cancer.^27^ Taken together, NIR‐PIT using Tra‐IR700 may provide a new treatment strategy even for breast cancers that have acquired resistance to trastuzumab‐mediated therapy.

In conclusion, our in vitro and in vivo results demonstrated that HER2‐targeted NIR‐PIT provides a high therapeutic outcome based on photochemical reaction‐caused stress in the cellular membrane, impairing its integrity. HER2‐targeted NIR‐PIT has been investigated in animal models of other cancers, showing high therapeutic effects.^3^, ^4^, ^5^, ^9^ HER2 is an attractive candidate for NIR‐PIT and is expected to be a novel treatment for breast cancer and other HER2‐positive cancers.

## AUTHOR CONTRIBUTIONS

All authors contributed to the study design. S. Yamashita, M. Kojima and N. Onda performed the data collection. S. Yamashita, M. Kojima, N. Onda and M. Shibutani performed the data analysis and interpretations of results. The first draft of the manuscript was written by S. Yamashita and all the other authors carried out the manuscript revising. All authors read and approved the final manuscript.

## CONFLICT OF INTEREST

S. Yamashita, M. Kojima, and N. Onda are employees of Olympus Corporation, which supported this work. M. Shibutani received a research grant from Olympus Corporation. The other authors have no potential conflicts of interest to disclose.

## Supporting information


Table S1.
Figure S1.
Figure S2.
Figure S3.

## Data Availability

The data that support the findings of this study are available from the corresponding author upon reasonable request.
