# Supplementary material for: Trastuzumab‐based near‐infrared photoimmunotherapy in xenograft mouse of breast cancer
Source: Cancer Med. 2022 Oct 18;12(4):4579–89. doi: 10.1002/cam4.5302 (PMC9972010; doi:10.1002/cam4.5302)
Supplement: Supplementary file 1 — Table S1. Figure S1. Figure S2. Figure S3. [file CAM4-12-4579-s001.docx]

**Supporting Information**

**Article title:** Trastuzumab-Based Near-Infrared Photoimmunotherapy in Xenograft Mouse of Breast Cancer

**Authors:** Susumu Yamashita, Miho Kojima, Nobuhiko Onda, Toshinori Yoshida and Makoto Shibutani

**The Supporting Information includes:**

**Supporting Information Figure S1–3**

**Supporting Information Tables S1**

**Corresponding authors: Makoto Shibutani (mshibuta@cc.tuat.ac.jp)**

**Table S1. Antibodies used in the present study**

|  | Abbreviated name | Manufacturer (City, State, Country) | Product # | Host species | Dilution |
| --- | --- | --- | --- | --- | --- |
| Antigen |  |  |  |  |  |
| human epidermal growth factor receptor 2 | HER2 | Cell Signaling Technology (Beverly, MA, USA) | 2165 | Rabbit | 1:100 |
| human epidermal growth factor receptor 2 | HER2 | DAKO (Carpinteria, CA, USA) | A048529 | Rabbit | 1:100 |
| Secondary antibody |  |  |  |  |  |
| Alexa Fluor 568 anti-Rabbit IgG (H+L) |  | Thermo Fisher Scientific (Walthan, MA, USA) | A10042 | Donkey | 1:500 |
| Alexa Fluor 647 anti-Human IgG (H+L) |  | Thermo Fisher Scientific | A21445 | Goat | 1:500 |

**
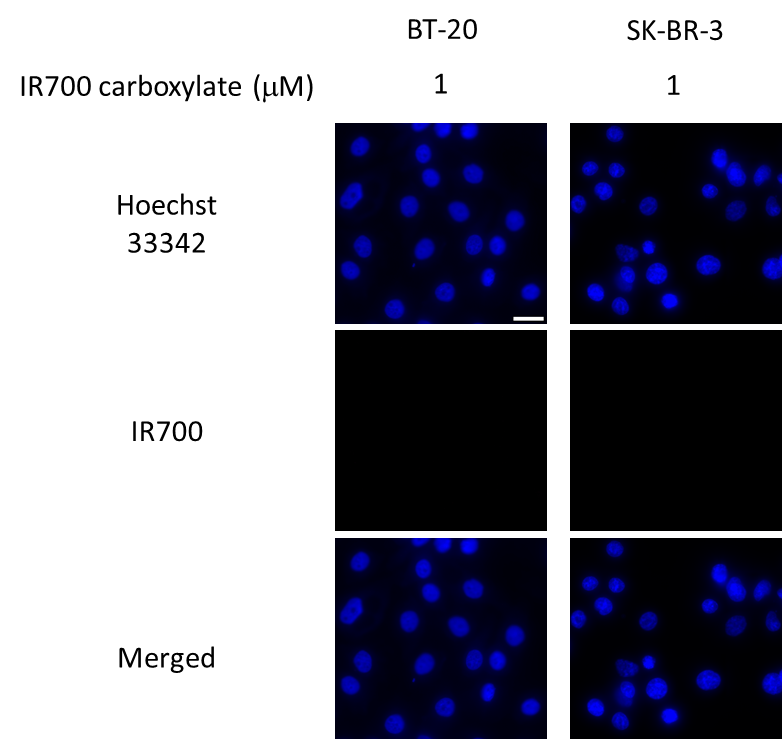
**

**Figure S1. Reactivity of IR700 on HER2-positive and negative breast cancer cell lines *in vitro*.** Fluorescence images of BT-20 and SK-BR-3 cell lines. IR700 fluorescence was not observed in both cells. Scale bar = 20 μm.

**
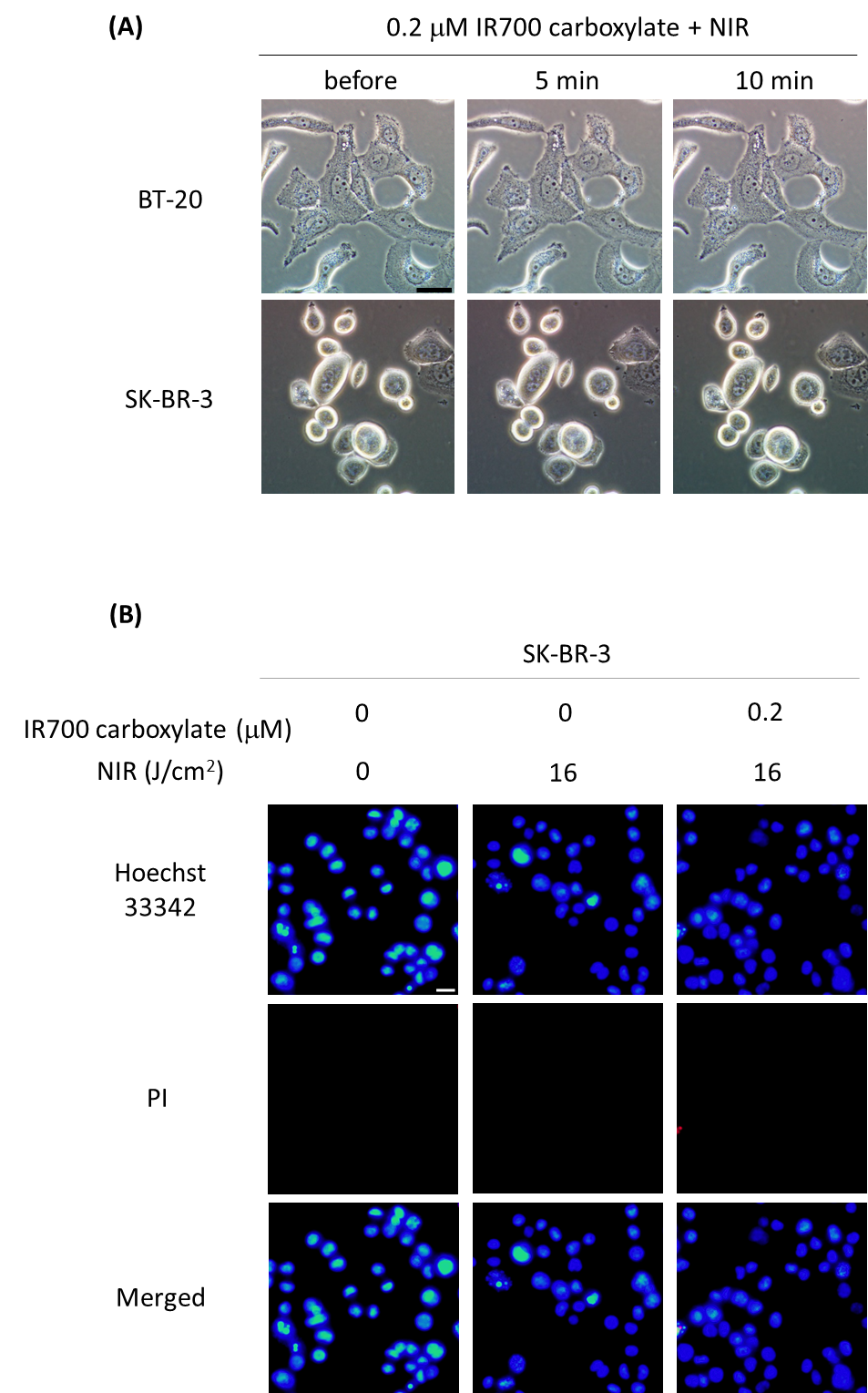
**

**Figure S2. Evaluation of cell damage by NIR light irradiation with IR700 in HER2-positive and negative breast cancer cell lines *in vitro*.** (A) Phase contrast images after NIR light irradiation in BT-20 and SK-BR-3 cell lines. IR700-treated BT-20 and SK-BR-3 cells did not show morphological changes after NIR light irradiation. Scale bar = 20 μm. (B) Double staining images of Hoechst and PI 1 day after NIR light irradiation. IR700-treated SK-BR-3 and BT-474 cells did not show PI signal. Scale bar = 10 μm

**
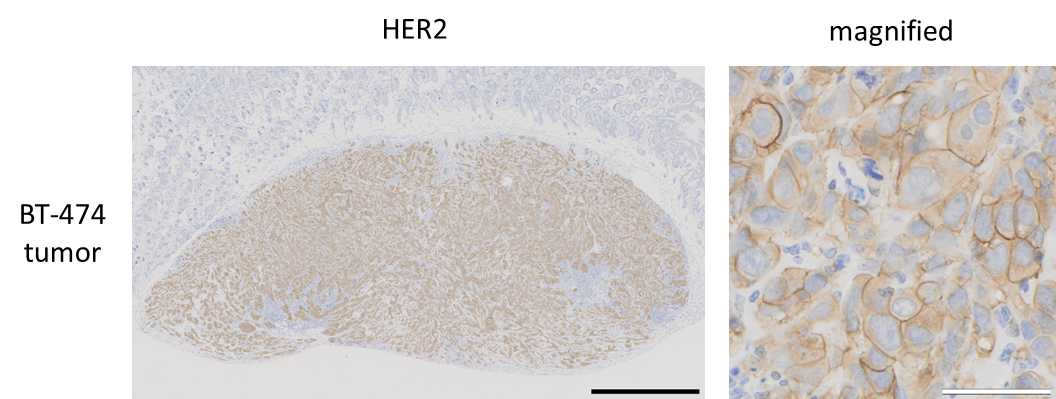
**

**Figure S3. HER2 immunoreactivity in the xenografted BT-474 tumor tissue.** BT-474 tumors showed diffuse cell membrane HER2 immunoreactivity. Sections were made from formalin-fixed, paraffin-embedded tumor samples. Deparaffinized sections were subjected to antigen retrieval treatment by microwaving in SignalStain EDTA unmasking solution (Cell Signaling Technology, Beverly, MA, USA) and quenching endogenous peroxidase by incubating in 0.3% hydrogen peroxide solution in absolute methanol. Immunohistochemical staining was performed using primary antibody against HER2 (Cell Signaling Technology) and Peroxidase-conjugated secondary antibody (Histofine Simple Stain MAX PO (R); Nichirei, Tokyo, Japan). Color development was performed with 3,3’-diaminobenzidine tetrahydrochloride/H_2_O_2_ in Tris-buffered saline (pH 7.6) as the chromogen. Nuclear counterstaining was conducted with hematoxylin and cover-slipped for microscopic examination. Black scale bar = 1 mm, White scale bar = 50 μm
